# Life-History Evolution on Tropidurinae Lizards: Influence of Lineage, Body Size and Climate

**DOI:** 10.1371/journal.pone.0020040

**Published:** 2011-05-13

**Authors:** Renata Brandt, Carlos A. Navas

**Affiliations:** Departamento de Fisiologia, Instituto de Biociências, Universidade de São Paulo, São Paulo, Brazil; University of Western Ontario, Canada

## Abstract

The study of life history variation is central to the evolutionary theory. In many ectothermic lineages, including lizards, life history traits are plastic and relate to several sources of variation including body size, which is both a factor and a life history trait likely to modulate reproductive parameters. Larger species within a lineage, for example tend to be more fecund and have larger clutch size, but clutch size may also be influenced by climate, independently of body size. Thus, the study of climatic effects on lizard fecundity is mandatory on the current scenario of global climatic change. We asked how body and clutch size have responded to climate through time in a group of tropical lizards, the Tropidurinae, and how these two variables relate to each other. We used both traditional and phylogenetic comparative methods. Body and clutch size are variable within Tropidurinae, and both traits are influenced by phylogenetic position. Across the lineage, species which evolved larger size produce more eggs and neither trait is influenced by temperature components. A climatic component of precipitation, however, relates to larger female body size, and therefore seems to exert an indirect relationship on clutch size. This effect of precipitation on body size is likely a correlate of primary production. A decrease in fecundity is expected for Tropidurinae species on continental landmasses, which are predicted to undergo a decrease in summer rainfall.

## Introduction

Life history traits are central to evolutionary theory because they relate to how evolution shapes growth and reproductive patterns, two key aspects of fitness. Because life-history traits are not necessarily independent, one important line of research focuses on how these traits relate to each other, and what evolutionary or phenotypic constraints may limit their expression [1]. Life history traits appear to be extremely plastic in many lineages of ectothermic vertebrates, including examples among amphibians [2] and fish [3]–[5]. Within squamates, several lizard species appear to display fix life history traits whereas others exhibit flexible patterns [6] . Several sources of variation in life history traits have been identified in the latter, including aspects of natural history and the action of physical parameters [7], [8]. Relevant natural history factors include lineage, foraging mode [9]–[13] and body size or body shape [9]–[13], which in turn may also be influenced by the environment.

Body size is both a life history trait itself and a factor likely to modulate diverse reproductive parameters. For example, lizard clutch size relates allometrically to body size [8], [12], [14] and to fecundity because, in species with variable clutch size, larger females tend to produce more eggs [6], [15]. In addition, clutch size may also increase with female body size across species [6], [15], [16], and several correlates of body size, including abdominal body volume, appear to play a role in this relationship [16]. Nevertheless, lizard clutch size may also be influenced by climate, independently of body size. Congeneric lizard species from different climate regimes (for example along latitudinal gradients) may differ in clutch size independently of differences in body size among populations [17]. Elements of climate known to influence lizard clutch size includes scope and pattern of climatic seasonality [6], and more pronounced seasonality favor larger clutch sizes [17]–[19].

Few studies focus on how climate influences life history in lizard lineages exhibiting environment-specific reproductive traits. These lineages may be particularly sensitive to climate change [20] and thus influenced by current global climatic trends. Therefore, we asked how body size and clutch size have responded to climate in a group of tropical lizards, and how these two variables relate to each other. To answer these questions we gathered climatic data from historical databases and studied body and clutch size mainly from preserved specimens catalogued in zoological collections. We focused on the sub-family Tropidurinae, a lineage that is morphologically and ecologically diversified, and that is distributed along a variety of contrasting habitats. Species representing this sub-family can be found in the Amazon Forest [21]–[24], the semi-arid Caatingas [25], [26] and the Cerrados (Brazilian Savannah) [27], three habitats with very different climate. Additionally, considerable information has been published on the ecology and natural history of the Tropidurinae, and specimens abound in Brazilian zoological collections. Furthermore, this lizard lineage displays large variation in body size and clutch size [28], [29]. We report on the relationships between body size, climatic conditions and clutch size in the Tropidurinae, using both published and new data collected from preserved specimens from zoological collections. We analyze the relationships between these variables using both traditional and phylogenetic comparative methods and test the hypothesis that larger species have larger clutch sizes, that clutch size and body size may be influenced by climate, and that size-independent effects of climate on clutch size can be detected.

## Results

### Climate

Data on environmental temperature and rain patterns were simplified using a principal component analysis that produced two main components, one clearly related to temperature (CC1) and another strongly related to rain patterns (CC2, Table 1, see Methods for details). The component CC1 (69.26% of total variance) was mainly influenced by annual mean temperature (AMT), mean minimum temperature (AMinT) and mean maximum temperature (AMaxT), but was also influenced by temperature records corresponding to the month in which gravid females were collected. These variables included mean temperature (MT), mean minimum temperature (MMinT) and mean maximum temperature (MMaxT). The component CC2 (23.23% of total variance) was related mainly to annual precipitation (AP) and monthly mean precipitation of records for gravid females (MP). The principal component scores retained for these climatic components, and corresponding to each one of the species included in this study, are available as supplementary supporting material.

**Table 1:** Scores of a Principal Component Analysis performed on climatic variables.

	Component	
climatic variable	1	2
Annual precipitation (AP)	0.429	0.864*
Annual mean temperature (AMT)	0.980*	0.125
Annual mean minimum temperature (AMinT)	0.926*	0.226
Annual mean maximum temperature (AMaxT)	0.943*	−0.007
Mean temperature of months with gravid females (MT)	0.982*	0.097
Mean minimum temperature of months with gravid females (MMinT)	0.879*	0.340
Mean maximum temperature of months with gravid females (MMaxT)	0.943*	−0.140
Mean precipitation of months with gravid females (MP)	−0.157	0.949*
**Eigenvalue (%variation explained)**	5.711 (69.26%)	1.689 (23.23%)

Note. Variables contributing most to each component are indicated by*.

### Body size and clutch size along the Tropidurinae

Both clutch and body size were variable within the Tropidurinae. Clutch size ranged from 1 egg (*Eurolophosaurus nanuzae, Plica plica, P. umbra, Tropidurus guarani, T. semitaeniatus, T. itambere, T. montanus, T. oreadicus and T. torquatus*) to 16 eggs (*Uranoscodon superciliosus*), and the size of gravid females ranged from 44.7 mm (*E. nanuzae*) to 151.0 mm (*P. plica) (a full list of clutch and body size means and ranges for all species studied is available on supporting material). Both traits were influence by systematic position within the Tropidurinae lineage (significant phylogenetic signal, see Table 2) under most models tested (clutch size exhibited marginally non-significant phylogenetic signal under constant branch lengths, P = 0.051, Table 2). Across the Tropidurinae lineage, species which evolved a larger female body size exhibited also larger clutch size (0.063<ß<0.067, p<0.01, phylogenetic models, Table 3). The climatic components did not influence clutch size among species (Table 3) but the climatic component CC2, which is associated with rain patterns, had a positive effect on body size across tropidurines (ß = 9.798, p<0.01, Table 4).*

**Table 2:** Parameters of phylogenetic signal for body size (snout-vent length  =  SVL) and clutch size.

trait	branches	MSEtree	MSEstar	K	P	ln likelihood tree	ln likelihood star
SVL	Constant	311.17	354.46	1.4194	0.021*	−89.5589	−90.9266
	Pagel	255.60	354.46	0.8479	0.004*	−87.4935	−90.9266
clutch size	Constant	2.6336	2.7859	1.0611	0.051	−39.4532	−40.0433
	Pagel	2.2850	2.7859	0.6885	0.005*	−37.9623	−40.0433

Note. Significant values are indicated by*.

**Table 3:** Comparisons of regression models testing the effects of body size (snout-vent length  =  SVL), climatic component 1 (CC1) and climatic component 2 (CC2) on clutch size.

		slope			F_1,16_							
branch length	model	SVL	CC1	CC2	Y	SVL	CC1	CC2	AIC	AICc	Ln L	d
none	OLS	0.05	−0.309	0.014	0.008	4.249	0.62	0.001	79.74	84.03	−34.87	
all equal 1	PGLS† §	0.067*	−0.246	0.118	0.626	17.82	1.02	0.203	68.57	72.86	−29.29	
	RegOU†	0.067*	−0.239	0.130	0.666	18.77	1.01	0.257	70.24	76.70	−29.12	1.105
Pagel	PGLS† §	0.063*	−0.163	0.177	0.752	13.64	0.53	0.471	69.90	74.19	−29.95	
	RegOU	0.063*	−0.177	0.161	0.770	13.18	0.58	0.372	71.55	78.01	−29.77	0.871

Note. Akaike Information Criterion (AIC; smaller is better) is computed as (2 * ln maximum likelihood) (2 * no. parameters). d is the restricted maximum likelihood estimate of the Ornstein-Uhlenbeck (OU) transformation parameter. Three linear regression models are compared: ordinary (nonphylogenetic) least squares (OLS), phylogenetic generalized least squares (PGLS), and regression in which the residual variation is modeled as an OU process (RegOU) along the specified phylogenetic tree.

*P<0.01.

† Model with best fit by smaller-is-better AIC criterion.

§ Model with best fit by smaller-is-better AICc criterion.

**Table 4:** Comparisons of regression models testing the effects of climatic component 1 (CC1) and climatic component 2 (CC2) on body size (snout-vent length  =  SVL).

		slope		F_1,17_						
branch length	model	CC1	CC2	y	CC1	CC2	AIC	AICc	Ln L	d
none	OLS† §	7.003	9.798*	474.87	3.882	7.6	171.121	173.787	−81.56	
all equal 1	PGLS	4.451	5.597	89.429	1.564	2.2	176.782	179.449	−84.39	
	RegOU†	7.003	9.798*	474.87	3.882	7.6	173.121	177.406	−81.56	2.60E−17
Pagel	PGLS	1.238	0.481	37.957	0.150	0.02	175.343	178.01	−83.67	
	RegOU†	7.003	9.798*	474.87	3.882	7.6	173.121	177.406	−81.56	2.60E−17

Note. Akaike Information Criterion (AIC; smaller is better) is computed as (2 * ln maximum likelihood) (2 * no. parameters). d is the restricted maximum likelihood estimate of the Ornstein-Uhlenbeck (OU) transformation parameter. Three linear regression models are compared: ordinary (nonphylogenetic) least squares (OLS), phylogenetic generalized least squares (PGLS), and regression in which the residual variation is modeled as an OU process (RegOU) along the specified phylogenetic tree.

*P<0.01.

† Model with best fit by smaller-is-better AIC criterion.

§ Model with best fit by smaller-is-better AICc criterion.

## Discussion

We found that body and clutch size in the Tropidurinae are heavily influenced by phylogenetic position, and that clutch size is a key component of fecundity. The dependence of body size on phylogenetic position has been previously reported for various lineages of animals [30], including lizards [30], [31]. Previous studies with tropidurines, however, did not report a significant effect of phylogeny on body size, in contrast to our findings [32], [33]. Because Kohlsdorf et al. [32] and Grizante et al. [33] analyzed male lizards and we studied females, the body size of male tropidurines may be more dependent on phylogenetic position than the body size of females. Overall, males and female lizards may differ in the degree of perceived phylogenetic niche conservatism, a condition that is suggested - not demonstrated - by phylogenetic signal [34] in the body size of gravid females. Conversely, lack of phylogenetic signal suggests that phylogenetic niche conservatism does not occur [34] in the body size of male Tropidurinae. Different selective pressures, therefore, may exist between sexes of tropidurine species, most of which are sexually dimorphic and display larger males.

Our results hint at an allometric and evolutionary relationship between body size and other life history traits [8], [12], [14], and specifically suggest that body size is the main correlate of clutch size among Tropidurinae lizards. Within tropidurines, at least in *Tropidurus spinulosus* and *T. torquatus,* females with larger body size produce eggs with larger mass and volume [27], [35], so clutch size seems associated with relative clutch mass (RCM) in this group of lizards. Whereas larger tropidurines tend to exhibit bigger clutch size, several species exhibit smaller clutch size than predicted by the body size – clutch size regression model. Examples include the Amazonian species *Plica plica, P. umbra* and *Uracentron flaviceps,* which rely on vertical locomotion. Vitt [22] analyzing the specific case of *Plica plica*, suggested that low clutch size and RCM are part of a pool of adaptations enhancing this type of locomotion. This finding is consistent with observations of clutch size in other groups of lizards, such as *Anolis* and most geckos, taxa in which natural selection favoring enhanced vertical locomotion may have led to reduced clutch sizes [36]. In the same paper, Vitt suggested also that the physical burden of a large clutch would not represent a disadvantage in other sit-and-wait species that do not use vertical locomotion [22], which are characterized by larger RCM [19]. These considerations receive independent support by the finding that gravid lizard females exhibit reduced endurance [37] and speed [38].

Some authors have suggested that smaller clutch sizes than predicted by regression models may occur in species associated with rock crevices, such as *Tropidurus semitaeniatus* [26], [39], [40]. Perhaps this is the case also for *T. mucujensis* and *T. erythrocephalus,* but given the small sample sizes available for these two species this hypothesis needs to be confirmed. Finally, the tropidurines encompass other species with clutch sizes that are also small, yet consistent with their body sizes (e.g., clutch size explained by the regression model). So, whereas habitat may play an important role in the evolution of lizard clutch size, it is not necessarily a factor in the evolution of small clutch sizes among tropidurines. For example, species such as *Eurolophosaurus nanuzae* exhibits small clutch sizes [28], yet it fits the predictions of a model based on body size. Thus, habitat type may be less important than formerly thought as a direct factor modulating clutch size and body size needs to be taken into account when analyzing environmental influences on clutch size across species within a lizard lineage.

**Figure 1:**
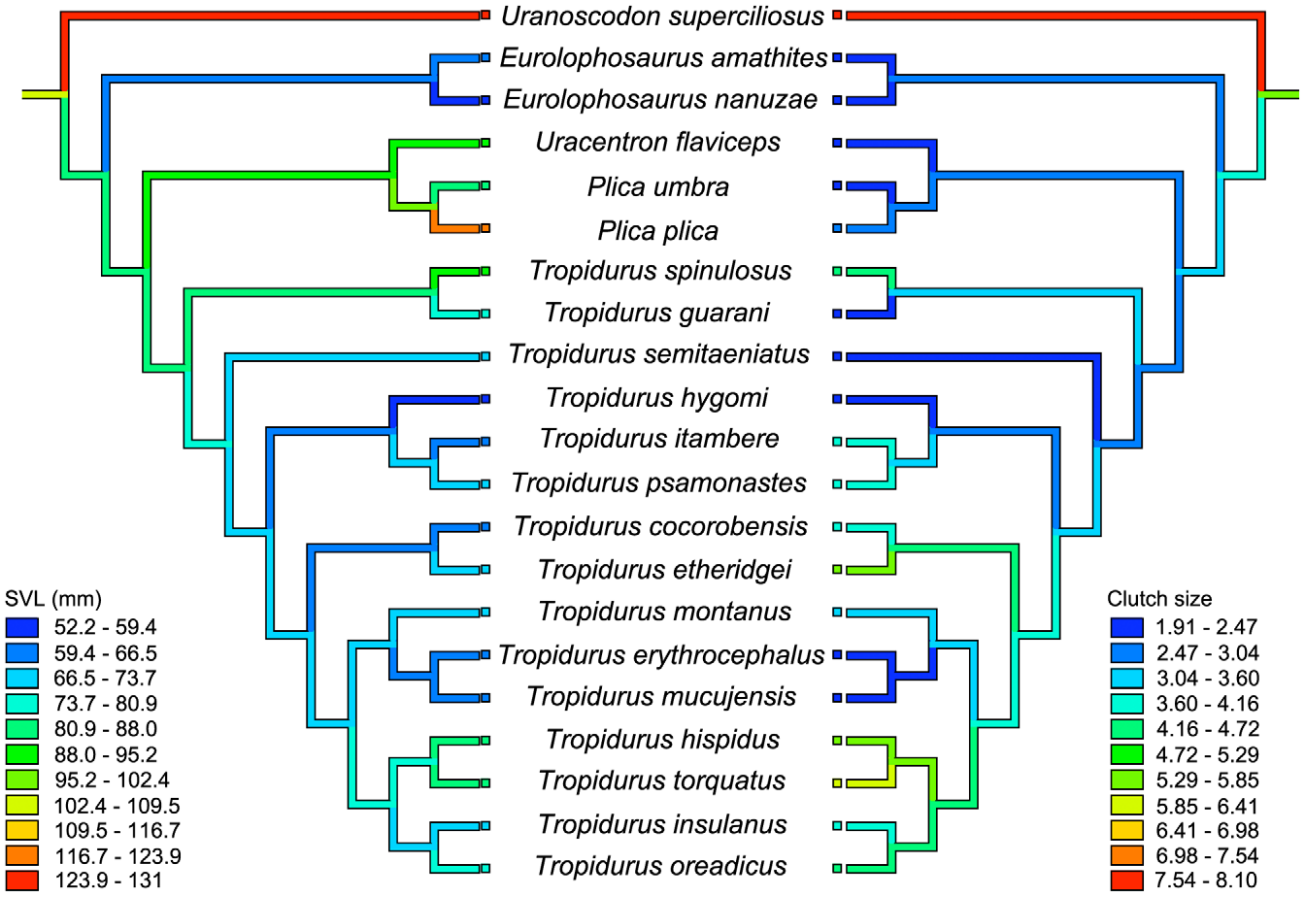
Topology of phylogenetic analysis and ancestral reconstruction of female body size and clutch size in the Tropidurinae. Topology used for phylogenetic analysis (based on [58]), together with parsimony reconstructions of ancestral body and clutch size in gravid females within Tropidurinae lizards.

Small clutch sizes may be a derived character for tropidurines, as has been suggested by Vitt et al. [24] and reinforced by Kiefer et al. [41]. Nevertheless, an ancestral reconstruction of clutch and body size for the Tropidurinae made with the data we have available (Figure 1) does not necessarily support this idea. We state this fully aware of the limitations of ancestral reconstruction techniques, especially when rates of evolutionary change are high (check [42] for a review) as seems the case for Tropidurinae lizards. Within this constraint, we noted that although small clutch size may have originated independently several times within the subfamily, the alternative view would take a similar amount of steps. Given this ambiguity, it is difficult to produce conclusive statements regarding whether smaller or larger clutches are ancestral or derived in this lineage (Figure 1).

Our data suggest that patterns of precipitation may exert an indirect effect on clutch size in the Tropidurines, perhaps via effects on primary production, but we do not know the underlying mechanisms or specific cause-effect relationships involved in this trend. Rain patterns, usually an overlooked component of climate, may influence lizard life history variation. For example, female body size covaries with precipitation in *Anolis mariarum* (Polychrotidae), presumably through effects on growth-rates [43]. In both *Aspidoscelis tigris* (Teiidae) [44] and *Sceloporus merriami* [45], [46] growth rates correlate with precipitation. In addition, rain patterns may influence reproductive traits in populations of the same lizard species. A comprehensive study with *Sceloporus merriami* shows that during wet years more arthropod preys are available and consumed, individual growth rates are higher and reproducing females are bigger. So, females exhibit greater size-specific fecundity in rainy than in dry years [45], [46]. These studies support a link between growth rates (and thus body size) and habitat production via climate effects that may also apply to some lineages of tropical lizards, for example the tropidurines.

We did not estimate primary production or food availability, so the above proposed relationship is speculative for the Tropidurinae but provides a likely explanation for a cause-effect relationship between clutch size and climate in this taxon. When rainfall is high, plant primary production is enhanced and provides primary consumers and its food webs with more food [47], [48]. For example, the body size of tenebrionid beetles is larger on habitats with higher rainfall regimes and therefore enhanced productivity [49]. Indeed, several groups of animals, from mammals to insects, exhibit larger body size in areas with higher precipitation [47]–[50]. Our study indicates that tropical lizards may conform to this pattern, and in at least one species (*Tropidurus itambere*) the number of reproductive females correlates with amount of rainfall [51]. These considerations are relevant for lizard conservation in the context of climate change. Most scenarios project reductions of rainfall in regions such as eastern Amazonia and Northeast Brazil [52]. According to our data, and independently of the mechanisms involved, the realization of such scenarios apparently would be paralleled by reduced reproductive output in Tropidurines, and perhaps in other lizard lineages. Conversely, Southern Brazil is likely to experience an increase in rainfall [52], which preliminary may suggest opposite effects. However, given that rain patterns may change as well (e.g. more extreme rainfall events [52]) conclusive statements are yet impossible. Finally, fecundity bears a relationship with yearly number of clutches and most Tropidurinae species are able to produce more than a clutch per year [53], but we are unaware of studies addressing how this latter variable bears dependence on precipitation patterns. In theory, yearly number of clutches could increase as rainfall decreases, as to compensate a reduction in clutch size. However, in the absence of studies supporting this possibility, we propose that a decrease in body size does decrease effective fecundity. The effects of changing rain profiles on the fecundity of lizards needs better understanding.

## Materials and Methods

### Body and Clutch Size

Body and clutch size were measured on lizards at three Brazilian herpetological collections: Museu de Zoologia da Universidade de São Paulo (MZUSP) - SP, Museu Paraense Emilio Goeldi (MPEG) - PA and Coleção Herpetológica da Universidade de Brasília (CHUNB) - DF (SP, PA and DF refer to Brazilian Federative Units). Snout-vent lenght (SVL) was used as body size estimation and was measured with digital calipers to the nearest 0.01 mm. Clutch size was estimated from the number of eggs or vitellogenic follicles found on dissected gravid females. All measurements were performed by RB. Additional data on body size and clutch size were obtained from the literature [22]-[24], [26]-[28], [35], [41], [51], , so that the final database comprised 21 Tropidurinae species out of the 50 species recognized by Frost et al. [59]. The sample size for each species studied is available as supporting material (see Table S1). Since often only few gravid females were available for a given species, data from different populations of the same species were pooled, so that species average values were entered in the analysis (see [60] for a similar approach).

### Climatic data

Climatic data were extracted from a historical database of daily records (1961−2009) available upon request from Instituto Nacional de Meteorologia (INMET), Brazil. We developed a criterion for choosing which climatic station better represented each locality using data from 42 climatic stations spread over a latitudinal gradient of more than 2800 km. On this data we performed a principal component analysis (PCA), using SPSS 16.0.1 for Mac OSX, and the principal components scores were regressed against latitude and altitude in a multiple regression analysis. Therewith, we predicted that climatic change of 100 m in altitude is equivalent to 100 km over the latitudinal gradient studied in Brazil. We considered the differences in altitude and latitude between the localities and the climatic stations available and calculated which would represent less change using our regression model.

For each locality/climatic station we chose among the climatic variables available those we judged best to characterize differences in local climate. We calculated yearly averages of the climatic variables AP, AMT, AMinT and AMaxT (see Results for full text on these acronyms). In additon, we produced another dataset containing monthly climatic data and therefore allowed for collecting data on the specific months with presence of gravid females: MP, MT, MMinT and MMaxT. These additional variables helped to investigate local differences in climate when females are more likely to be gravid. Climatic values representing a species were pooled in the same manner described above for populations. We reduced the climatic variables using PCA, so that they would not display multicolinearity. Components with eigenvalues >1 were retained and their scores were used in subsequent analysis.

### Analysis

A traditional way to study evolutionary correlations between traits is to make interspecific comparisons, which are best analyzed under a phylogenetic perspective. The main reason is that phylogenetic approaches account for common ancestry, which is responsible for shared similarities exhibited by closely related species. Accordingly, for data analysis we built a topology (Figure 1) based on Frost et al. [59], which incorporates molecular and morphological data to enhanced previous phylogenetic hypothesis for the Tropidurinae sub-family [61], [62]. Estimates of branch length, such as divergence time, genetic distance or any other metric proportional to the expected variance for the evolution of each analyzed trait, are unavailable. Consequently, we tested four different types of arbitrary branch lengths following the diagnostics proposed by Garland et al. [63]: all equal 1 (Constant), Grafen [64], Pagel [65] and Nee (cited in [66]). Only Constant and Pagel arbitrary branch lengths passed the diagnoses criteria and were therefore both used in our subsequent analysis. We used Mesquite v2.74 [67] with PDAP:PDTREE v1.15 [68] module for Mac OSX to manipulate trees and branch lengths as well as to examine diagnostic plots of independent contrasts.

We tested (1) the effects of body size and climatic components on clutch size; and (2) the effects of climatic components on body size by using linear regression models implemented via Regressionv2.m in MATLAB 7.6 (R2008a) for Mac OSX. Three types of models were examined: (a) ordinary least squares (OLS), which is a traditional non-phylogenetic regression that assumes a star phylogeny in which all species are equally unrelated, (b) phylogenetic generalized least squares (PGLS), which is functionally equivalent to Felsenstein's [69] phylogenetically independent contrast method [70] and assumes that residual variation between species is correlated through an evolutionary process along the specified phylogenetic tree similar to a Brownian-motion (topology and branch lengths); (c) and a phylogenetic regression under an Ornstein-Uhlenbeck process (RegOU), similar to PGLS with the difference that it allows transformation of branch lengths under a stabilizing selection evolutionary model while estimates simultaneously the strength of phylogenetic signal in the residual variation and the regression coefficients using the parameter *d* [71]. The parameter *d* returns a measure of phylogenetic signal — no phylogenetic signal when d = 0, existent and significant phylogenetic signal when d is significantly greater than zero [30], [71]. These three models form a continuum between assuming a star phylogeny with no hierarchical structure (OLS), a phylogeny that was specified by the user (PGLS) and a phylogeny that can exhibit intermediate values between the star and the specified hierarchical phylogeny (RegOU). We also performed the randomization test for phylogenetic signal of Blomberg et al. [30]. Generally, whether conventional or phylogenetical statistics should be used to interpret the results depends on whether the analyzed traits exhibit phylogenetic signal. We report results from both approaches (traditional and phylogenetic) together with the Akaike Information Criterion (AIC), a heuristic indicator of model support, in which AIC  =  (-2 * ln maximum likelihood) + (2 * n° of parameters). Because sample size was not large, we also computed the AICc [72], which in all cases gave results consistent with AIC. We followed Burnham and Anderson [72] and considered that the best-fit model is that with the lowest AIC. As a rule of thumb, models with AIC within 2 units of the best-fit model were considered to have substantial support. All variables included in the best-fitting models were statistically significant (P < 0.05) via partial-F tests. The MS-DOS computer program PDDIST [63] was used to build the phylogenetic variance-covariance matrix used in the analysis [71].

## Supporting Information

Table S1 List of studied species, means and ranges for clutch size (CS) and snout vent lenght (SVL) and scores for the climatic components CC1 and CC2 used in the analysis.
(PDF)
