# Supplementary material for: Life-History Evolution on Tropidurinae Lizards: Influence of Lineage, Body Size and Climate
Source: PLoS One. 2011 May 13;6(5):e20040. doi: 10.1371/journal.pone.0020040 (PMC3094402; doi:10.1371/journal.pone.0020040)
Supplement: Table S1 — List of studied species, means and ranges for clutch size (CS) and snout vent lenght (SVL) and scores for the climatic components CC1 and CC2 used in the analysis. (PDF) [file pone.0020040.s001.pdf]

Table S1 - List of studied species, means and ranges for clutch size (CS) and snout vent lenght (SVL) and scores for the climatic components CC1 and CC2 used in the analysis.

Species	n	CS (range)	SVL (range)	CC1	CC2	Source
U. superciliosus	18+28	7.54 (3-16)	123.86 (104.1-143)	0.69221	1.72601	[1] and this study
E. amathites	4	2	59.4 (57.8-61)	0.77004	-2.04981	this study
E. nanuzae	90	2.06 (1-3)	52.2 (44.7-60.3)	-2.00146	0.53538	[2]
U. flaviceps	5	2	91.8(88-96)	–	–	[3]
P. umbra	10+25	1.9 (1-2)	85.36 (72.8-93)	0.8295	1.4441	[4] and this study
P. plica	16+8	3.01 (1-5)	121.41 (88-151)	0.80565	1.22856	[5] and this study
T. spinulosus	21+35+26	4.5 (3-7)	90.15 (72-110.2)	-0.49363	-0.2092	[6-8]
T. guarani	5	2 (1-3)	76.9 (69.34-84.53)	0.96433	-0.83081	this study
T. semitaeniatus	86	2 (1-3)	72.4 (58-83)	0.7832	-0.99499	[9,10]
T. hygomi	7	2	55.14 (50.8-61.11)	0.69006	0.64964	this study
T. itambere	35+33	3.75 (1-8)	64.33 (52.3-67.15)	-0.87796	0.34935	[11] and this study
T. psamonastes	3	4	73.06 (72.34-73.78)	0.32155	-1.4964	this study
T. cocorobensis	4	4	62.3 (60.74-66.1)	-1.11465	-1.28529	this study
T. etheridgei	45+59	5.58 (3-9)	67.10 (61-79)	-0.06091	-0.17718	[6,12]
T. montanus	52	3.48 (1-6)	69.79 (56.5-82.6)	-2.01467	0.67611	[13]
T. erythrocephalus	4	2	63.1 (60.74-69.2)	-1.16491	-0.60222	this study
T. mucujensis	3	2	65.2 (61.53-67)	0.04702	-0.37238	this study
T. insulanus	14	3.92 (3-5)	68.21 (62.69-74)	1.17216	0.2265	this study
T. oreadicus	92	4.05 (1-8)	72.41 (58.1-94)	0.49312	0.73022	this study
T. hispidus	90+25	7.3 (2-14)	81.55 (68.3-107)	0.83909	6.40E-04	[9,10] and this study
T. torquatus	56+31	3.93 (1-10)	82.69 (55.2-112)	-0.67973	0.45178	[14] and this study

References for the sources

1. Howland JM, Vitt LJ, Lopez PT (1990) Life on the edge: the ecology and life-history of the tropidurine iguanid lizard *Uranoscodon superciliosum*. Canadian Journal of Zoology 68: 1366-1373.
2. Galdino CAB, Assis VB, Kiefer MC, Van Sluys M (2003) Reproduction and fat body cycle of *Eurolophosaurus nanuzae* (Sauria; Tropiduridae) from a seasonal montane habitat of Southeastern Brazil. Journal of Herpetology 37: 687-694.
3. Vitt LJ, Zani PA (1996) Ecology of the elusive tropical lizard *Tropidurus* [equals *Uracentron*] *flaviceps* (Tropiduridae) in lowland rain forest of Ecuador. Herpetologica 52: 121-132.
4. Vitt LJ, Zani PA, Avila-Pires TCS (1997) Ecology of the arboreal Tropidurid lizard *Tropidurus (=Plica) umbra* in the Amazon region. Canadian Journal of Zoology 75: 1876-1882.
5. Vitt LJ (1991) Ecology and life-history of the scansorial arboreal lizard *Plica plica* (Iguanidae) in Amazonian Brazil. Canadian Journal of Zoology 69: 504-511.
6. Vitt LJ (1991) An introduction to the ecology of cerrado lizards. Journal of Herpetology 25: 79-90.
7. Cruz FB, Teisaire E, Nieto L (1997) Reproductive biology of the lizard *Tropidurus spinulosus* in the chaco of Salta, Argentina. Studies on Neotropical Fauna and Environment 32: 28-32.
8. Martori R, Aun L (1994) Aspects of the ecology of a population of *Tropidurus spinulosus*. Amphibia-Reptilia 15: 317-321.
9. Vitt LJ, Goldberg SR (1983) Reproductive ecology of 2 tropical iguanid lizards - *Tropidurus torquatus* and *Platynotus semitaeniatus*. Copeia 1983: 131-141.
10. Vitt LJ (1993) Ecology of isolated open-formation *Tropidurus* (Reptilia, Tropiduridae) in amazonian lowland rain-forest. Canadian Journal of Zoology 71: 2370-2390.
11. Van Sluys M (1993) The reproductive-cycle of *Tropidurus itambere* (Sauria, Tropiduridae) in southeastern Brazil. Journal of Herpetology 27: 28-32.
12. Cruz FB (1997) Reproductive activity in *Tropidurus etheridgei* in the semiarid chaco of Salta, Argentina. Journal of Herpetology 31: 444-450.
13. Van Sluys M, Mendes HMA, Assis VB, Kiefer MC (2002) Reproduction of *Tropidurus montanus* Rodrigues, 1987 (Tropiduridae), a lizard from a seasonal habitat of south-eastern brazil, and a comparison with other *Tropidurus* species. Herpetological Journal 12: 89-97.
14. Wiederhecker HC, Pinto ACS, Colli GRR (2002) Reproductive ecology of *Tropidurus torquatus* (Squamata : Tropiduridae) in the highly seasonal Cerrado biome of Central Brazil. Journal of Herpetology 36: 82-91.
